# Five-year fidelity assessment of an evidence-based parenting program (GenerationPMTO): inter-rater reliability following international implementation

**DOI:** 10.1186/s12913-023-09611-4

**Published:** 2023-06-05

**Authors:** Margrét Sigmarsdóttir, Melanie M. Domenech Rodríguez, Abigail Gewirtz, Laura Rains, Jolle Tjaden, Marion S. Forgatch

**Affiliations:** 1 School of Education, University of Iceland, Reykjavík, Iceland; 2 Department of Psychology, Utah State University, Logan, USA; 3 Department of Psychology, Arizona State University, Tempe, USA; 4 Implementation Sciences International, Inc., Eugene, USA; 5 PI Research, Duivendrecht, Netherlands; 6 Oregon Social Learning Center, Eugene, USA

**Keywords:** Implementation, Fidelity, Evidence-based parenting program, GenerationPMTO

## Abstract

**Background:**

Implementing evidence-based programs in community service settings introduces the challenge of ensuring sustained fidelity to the original program. We employ a fidelity measure based on direct observation of practitioners’ *competence* and *adherence* to the evidence-based parenting program (EBPP) GenerationPMTO following installation in national and international sites. Fidelity monitoring is crucial, especially when the program purveyor transfers administration of the program to the community as was done in this case. In previous studies, the Fidelity of Implementation rating system (FIMP) was used to evaluate practitioners’ fidelity to the GenerationPMTO intervention in six countries following implementation showing high levels of adherence up to 17 years post certification. Other studies showed FIMP to have predictive validity. The present study provides inter-rater reliability data for this fidelity tool across teams of the purveyor, Implementation Sciences International, Inc./ISII, and national and international sites over a five-year period.

**Methods:**

Data assess inter-rater reliability in terms of percent agreement and intraclass correlation (ICC) for the purveyor’s two fidelity teams and the fidelity teams in seven implementation sites.

**Results:**

Results report stable good to excellent levels of inter-rater reliability and ICCs as well as good attendance at fidelity meetings for all fidelity teams.

**Conclusions:**

This observational method of assessing fidelity post implementation is a promising approach to enable EBPPs to be transferred safely from purveyors to communities while maintaining reliable fidelity to the intervention.

Implementing evidence-based programs in community service settings presents the challenge of ensuring sustained fidelity to the core components and processes that produced positive outcomes in carefully controlled settings [1–3]. Fidelity is important because failure to deliver interventions as designed by their developers risks failure to replicate positive outcomes [4–6] and could even possibly lead to iatrogenic effects [1]. Definitions of implementation fidelity vary. For example, some specify that fidelity incorporate adherence, differentiation, and competence [2, 7], whereas Durlak and DuPre [5] include dosage, quality, participant responsiveness, monitoring of control/comparison conditions, program reach, and adaptation in the definition. This paper evaluates the reliability of a fidelity measure based on direct observation of practitioners’ *competent adherence* to GenerationPMTO following installation in national and international sites. GenerationPMTO is an evidence-based parenting program (EBPP) originally developed to treat children’s behavior problems and promote healthy development [8–10]. Our fidelity measure is the Fidelity of Implementation rating system (FIMP) [11].

Fidelity monitoring supports the successful transfer of evidence-based programs from the carefully controlled environments where they were developed and tested to the real-world conditions of community practice. Practice of the intervention can gradually change from the specified program and compromise outcomes [5, 12]. This possibility of drift makes regular evaluation of fidelity an essential part of conducting psychological intervention research and clinical practice [13]. Valid fidelity assessment enables intervention and implementation researchers to understand implementation failures or “voltage drop” [14] and determine whether an implementation effort has failed because the intervention was ineffective in the new setting or because of a fidelity failure [4, 15]. Although systematic monitoring of practice in real-world circumstances can promote sustained program fidelity and positive outcomes, such practice is rare, with a dearth of research for evidence-based practices and the methodology for fidelity assessment [16]. Valid assessment must include that fidelity raters maintain inter-rater reliability through regular monitoring and calibration with predetermined criteria [17].

## Measuring program fidelity

Historically, parent training interventions have used client, peer, and self-evaluation ratings, and direct observation methods to evaluate fidelity [7]. Although ratings made by non-independent informants tend to yield larger effect sizes, they can be subject to social desirability and other biases [15, 18]. A recent meta-analysis of fidelity examined relationships among adherence, competence, and outcomes; measures based on direct observational approaches were preferred to report provided by non-independent informants. Non-independent reports had a 66% risk of bias in reliability; observational measures, on the other hand, did well at meeting inter-rater reliability thresholds [15].

A recent systematic review of fidelity measures in parenting programs found that adherence (the degree to which a practitioner uses specified procedures) was measured alone 48.3% of the time and competence (the practitioner’s skill in delivery) alone 48.3% of the time; 34.4% measured competent adherence and 13.9% measured competence and adherence [19]. GenerationPMTO endorses the perspective that adherence requires competent delivery [2] and thus evaluates competent adherence using the Fidelity of Implementation Rating System (FIMP) [11]. For observational methods to produce reliable and valid outcomes, several issues must be addressed, including the following: predictive validity, what is observed, how much is sampled, who the coders are, training of coders, and regular monitoring of interrater reliability.

### Predictive validity

Reports of the predictive validity of fidelity measurement are limited [19, 20]. Two programs that report predictive validity for their measures are the Family Check-Up (FCU) [21] and GenerationPMTO [9]. The FCU fidelity measure scores videotaped observations of intervention sessions that combine motivational interviewing and parent-training. Findings showed that high fidelity predicted greater caregiver engagement, which in turn predicted improvement in parents’ positive behavior support one year later, which in turn predicted reductions in children’s problem behavior two years later [22]. A study examining fidelity drift in FCU over four years found a slight decline in fidelity, which was associated with less improvement in caregiver reported problem behaviors [12]. For GenerationPMTO, observations of sessions in an efficacy trial were replicated during a community practice trial, and both studies found that high fidelity observed during intervention predicted positive change in pre/post observations of parenting practices [23]. A community study in Norway found that fidelity assessed three times during intervention predicted improvement in pre/post parent-reported child behavior [24].

### Other measurement issues

Durlak and Dupre [5] emphasize that fidelity measures must specify how well theoretically important components of the intervention are delivered. GenerationPMTO identifies five core components: skill encouragement, limit setting, monitoring, problem solving and positive involvement. Sessions assessed for fidelity by the FIMP code are the contingency-based components skill encouragement and limit setting [25].

The amount of sampling of an observation is a concern. For research purposes or practitioner certification, whole sessions may be appropriate. In widescale implementation, sampling core component sessions addresses cost effective needs and session segments appear to be sufficient to evaluate fidelity levels that replicate outcomes produced in more controlled settings [23, 24]. The FIMP samples sessions in two ways. For certification purposes, four sessions from two core components are viewed in their entirety. For training, coaching, and sustained cross-site reliability assurance, 10 to 15-min session segments are scored. The segments are selected by trained assistants who spot-check tapes of core components that include a teaching activity such as debriefing home practice, role playing, or brainstorming [23].

Another fidelity issue involves who scores the intervention sessions: students, supervisors, skilled practitioners of the method, or non-practitioners who are trained to reliability in the coding system. Smith and colleagues [22] employed a heterogenous set of reliable coders that included trained practitioners and nonpractitioners. They concluded that trained practitioners of the method are most suited to reliably assess competent adherence. For GenerationPMTO, FIMP coders are certified practitioners [11].

The quality of the therapeutic alliance is widely asserted to be relevant to clinical change [26]. Smith et al. [22] suggest that although this relationship may be a necessary aspect of intervention, it is not sufficient to ensure behavior change. Indeed, in a community study of GenerationPMTO in Norway, FIMP fidelity and working alliance [27] were assessed simultaneously three times during intervention; high levels of FIMP fidelity predicted reductions in parent-reported externalizing behavior, whereas strong working alliance reported by parents was associated with no change in child behavior [24]. This is not to say that therapeutic alliance is irrelevant but rather that it needs to be paired with other characteristics. Some FIMP code categories incorporate therapeutic alliance within their definition. For example, in the category *overall development,* practitioners are rated for quality of their relationship with clients and their capacity to tailor methods to address family or contextual situations, and the category *structure* requires responsiveness to the clients’ issues [11].

“Like any assessment device, an observational system should be evaluated in terms of the various psychometric properties subsumed under the traditional notions of reliability and validity” (p.11) [17]. We define coder reliability as the degree to which coders score behaviors in accordance with a predefined criterion, in this case the five FIMP categories as defined in the FIMP manual [11]. Inter-rater reliability across diverse implementation sites is the *sine qua non* of successful fidelity monitoring. A key priority of GenerationPMTO is ensuring that all FIMP coders score their observations using the metrics specified in the manual. Sustaining reliable assessment across implementation sites, time, and contexts requires a structure that supports the training and retraining of coders and the monitoring of coders’ scores. The present report provides five years of outcome data for the inter-rater reliability of the purveyor’s FIMP teams (*n* = 2), and the FIMP teams of seven implementation sites.

## GenerationPMTO

A progenitor in the field of evidence-based parenting programs, GenerationPMTO provides a range of preventive and clinical interventions for families with children and youth between the ages of 2–18 [9, 10, 28, 29]. GenerationPMTO training is carried out by ISII, a non-profit affiliate of the Oregon Social Learning Center. The program, developed to address child and adolescent behavior problems and promote healthy development, is provided through group, individual, and telehealth delivery systems. Based on coercion theory and the social interaction learning (SIL) model, a core assumption is that problem behavior lies not only within the child or adolescent but also in the social environment [30]. Since disrupted parenting practices are the presumed mediators of children’s behavior problems, the intervention focuses on strengthening parenting. Thus, parents are the agents of change in this family-oriented program. One goal is to reduce coercive family interactions (i.e., aversive behaviors, negative reciprocity, escalation, and negative reinforcement). The other primary goal is to increase positive parenting practices, which include *skill encouragement, limit setting, monitoring, problem solving, positive involvement*, emotion identification and regulation, active communication, effective directions, and promoting school success. The italicized skills are deemed the core components of the intervention.

In the course of its 50-year history, the program has been developed and tested through an iterative process among theory, practice, and research. Practice in multiple communities has taught that the intervention must be tailored and delivered sensitively across multiple contexts and populations. Adaptations have been tested experimentally yielding positive results [31–36]. Widescale implementation of GenerationPMTO has been carried out in cities, states, and countries in North and South America and Europe. Detailed information about GenerationPMTO, its theoretical background, research, practice, and implementation can be found in multiple publications [9, 28, 29, 37–41].

### Implementation strategy: full transfer

GenerationPMTO employs a full transfer approach to implementation [23, 42]. Full transfer involves more than the train-the-trainer approach. In a train-the-trainer framework, trainees become trainers who can then become local experts. In the full transfer approach, the process begins when ISII trains the first group of community practitioners to certification. From this group, those with high levels of skill and commitment are selected for training to certification as trainers, coaches, and fidelity coders. Once a local team grows in their abilities to carry out the multiple layers of GenerationPMTO implementation, then a governing authority is established, which oversees all GenerationPMTO activities within the site and maintains communication with the purveyor team at ISII. Sites develop their own FIMP teams of certified coders to monitor the fidelity of their providers (e.g., country, state, city, agency). The purveyor requires that fidelity teams meet regularly to maintain inter-rater reliability based on intervention sessions within their site and pass an annual reliability test. The goal is full transfer with model fidelity. Through full transfer, the community gradually assumes full responsibility for all activities involved in practice: training, coaching, certification, and continuous monitoring of fidelity and outcomes. Each year, sites pay a nominal fee that covers use of a secure portal for reliability testing and consultation. Sites’ fidelity teams are funded in various ways (e.g., block grants, state/national government, or local agency budgets).

Two studies evaluated this full transfer approach by examining the fidelity at certification of several generations of practitioners trained by the community. In the Norwegian implementation, program adoption and fidelity were sustained across seven generations for 17 years following the introduction of GenerationPMTO with a mean certification rate of 94% [37]. In a study of implementations in Iceland, Denmark, and the Netherlands, program adoption and fidelity were sustained with six generations in Iceland, eight in Denmark, and four in the Netherlands, with a mean certification rate in each country of at least 80% [41]. These studies indicate that adopting communities can sustain the full transfer approach.

### This study

In this article, we describe a method of collecting fidelity data based on direct observation of intervention. We empirically examine five years of data that assess the participation and inter-rater reliability of FIMP coders from the purveyor’s two teams (i.e., ISII-1, and ISII-2) and seven national and international implementation sites. Prior studies showed that the program was sustained for several years with high fidelity in four European sites when assessing therapists´ fidelity at GenerationPMTO certification [37, 41]. The goal of this study is to document the strategy to establish the inter-rater reliability of fidelity ratings for the purveyor´s teams and all implementation sites over a five-year period. Findings will provide valuable information about how this assessment system can help programs be transported effectively to community services across international contexts We address two questions: (1) Do the purveyor’s two FIMP teams maintain high levels of attendance and stable acceptable levels of inter-rater reliability, and (2) Do the implementation site teams maintain high levels of attendance and stable acceptable levels of inter-rater reliability?

## Method

### Participants

Participants are fidelity coders from the purveyor’s two FIMP teams (ISII-1 [*n* = 5] and ISII-2 [*n* = 6]) and the teams from seven implementation sites (*n* = 46). All FIMP coders are certified GenerationPMTO specialists who are also certified fidelity coders.

The ISII-1 FIMP Team, which is the consensus team, has been meeting to calibrate fidelity since 2010 with the same five people: the ISII Fidelity Director, who serves as the team leader; the ISII Director of Implementation and Training; the treatment developer; and two senior ISII Mentors who are also researchers. At each ISII-1 Team meeting, FIMP leaders from one or two of the implementation sites participate on a rotating basis. The team establishes consensus scores that are then used by FIMP coders from the implementation sites as standards for the annual reliability test.

The ISII-2 FIMP Team is under the direction of the ISII Fidelity Director to rate the work of practitioners within ISII and across the implementation sites. Team members are mentors who score a mélange of implementation activities, including: certifying and recertifying practitioners, coaches and trainers from the implementation sites; scoring and providing feedback to trainees; scoring the work of the purveyor’s trainers and coaches; and providing extra support for FIMP coders who become unreliable. The number of participants varies.

Implementation sites are required to maintain their own team of fidelity coders to score session material within their sites, maintain within-site reliability, and complete an annual reliability test that requires coding of at least three “test spots” provided by the ISII-1 Team. In addition, fidelity leaders from each implementation site join about two ISII-1 FIMP meetings each year (i.e., about 10 of the 11 meetings include a site FIMP lead). During these meetings, the group typically scores a clip from that site. These meetings provide an opportunity for cross-fertilization of knowledge as well as ensuring ongoing reliability between sites and ISII. Tricky coding issues are raised, and these sometimes result in changes to the FIMP manual; discussions and decisions are fed back to the implementation site through its FIMP leader. The seven implementation site teams are from six countries (i.e., Canada, Denmark, Iceland, the Netherlands, Norway, United States) with two sites within the US (i.e., Kansas, Michigan). The number of participants in each team varies across site and year (ranging from 3–15) depending on factors like number of active therapists and workload on site. All participating sites were contacted by email, informed about the study, and asked to provide their permission for use of the deidentified data. No formal ethical process took place since all material is deidentified and located on the same HIPAA-secure database owned by the purveyor.

### Measure

The Fidelity of Implementation Rating System [11] is scored from video recordings of intervention sessions. FIMP has five dimensions: (a) knowledge: understanding of the model and principles; (b) structure: use and flow of an agenda while being responsive to family’s needs; (c) teaching: balancing didactic instruction with active approaches such as role playing and problem solving; (d) process: use of sophisticated clinical techniques that create a safe and balanced learning environment; and (e) overall development: relationship between therapist and family and the extent to which families show growth, engagement and satisfaction. Each dimension is scored on a scale that ranges from 1 (*no evidence of competence*) to 9 (*exemplary*). These scores are grouped generally into the superordinate domains *needs work* (1–3), *acceptable* (4–6), and *good work* (7–9).

The FIMP measure is used for several purposes: to certify and recertify GenerationPMTO specialists, group facilitators, trainers, and coaches; to assess progress during training; to maintain FIMP reliability; to assess fidelity during intervention; and to prevent fidelity drift within and across implementations sites. When rating for certification/recertification of practitioners, reliable FIMP coders view four complete intervention sessions with a minimum of two families or groups. The session topics are introducing and troubleshooting encouragement and introducing and troubleshooting limit setting. For recertification, the general rule is that one yearly session from each PMTO practitioner is viewed from one of these topics. Candidates must achieve a mean score of 6 or higher with no score lower than 4 on any dimension. Scores with a mean of 7.6 or higher receive an *exemplary pass*.

#### Consensus score

The consensus scores originate in the ISII-1 Team meetings where the five core members of the team are present as well as one or two FIMP leaders/representatives from the countries or sites for which the video to be coded is obtained. Site team representation ensures that the context of each site is taken into consideration as the consensus scores are generated. Prior to the ISII-1 meetings, coders independently record their scores and notes on the website and later arrive at the meeting. Their notes specify how their scores are based on the FIMP manual in each of the five dimensions (typically 3–5 comments per FIMP dimension).The FIMP manual serves as the criterion determining appropriate code category and rating score. Consensus decisions are in keeping with specifications in the manual. About 30 to 60 min prior to the FIMP team meeting, the Fidelity Director releases scores for all of those who have provided ratings for the current meeting. This allows the team to see each rater’s notes and the spread in scores. The Fidelity Director can then lead the meeting efficiently by encouraging coders to comment, grouped according to their scores. It is typical for each member to comment on each FIMP dimension and provide a rationale for their score. In the end, the team agrees on a final score to capture each dimension based on criteria specified in the manual.

The ISII-1 consensus meetings establish and continually maintain the construct validity of the FIMP measure. This group of recognized subject matter experts discuss competent adherence in relation to an established benchmark, the FIMP manual [11], in monthly meetings and focus on the fundamental question regarding the suitability of indicators (i.e., GenerationPMTO practitioners’ implementation of knowledge, structure, teaching, process, and overall development) to assess the construct being measured (i.e., fidelity). The attention to contextual factors and inclusion of diverse stakeholders in the process of maintaining construct validity also promotes external validity. FIMP’s already established predictive validity [23–25] supports the value of these processes. The current manuscript examines FIMP’s five-year inter-rater reliability across sites to provide further support for the feasibility of employing an observational tool to prevents fidelity drift from practice of the intervention.

### Procedures

Each FIMP team has a leader, a reliable coder who makes assignments, leads team meetings, monitors reliability, and conducts team training and retraining. Prior to meetings, team members receive an assignment to score a FIMP spot, which is a 10 to 15-min video segment from an intervention session. FIMP spots are chosen as meaningful exemplars from practitioners’ work in the implementation site. Prior to the meeting, coders enter their scores and written rationales into FIMP Central, a HIPAA-secure database where data are uploaded, stored, viewed, and monitored. Ratings are masked until all ratings have been submitted. Coders score each FIMP dimension and supply a few bullet points of narrative to support their scores. Scoring FIMP spots typically requires three to four times the duration of the spot. Meetings are highly structured, with the team leader moderating the discussion, particularly when disagreements occur; this, together with referral to the detailed fidelity manual, provide the necessary framework to assess fidelity and reach consensus. Fidelity meetings last approximately 1.5 h and follow a structured agenda. During the meeting, team members view each other’s scores, discuss each category and the scores, and evaluate reliability.

Adequate inter-rater agreement is achieved when a coder has 80% of scores within one point of the consensus score. A passing score for coder certification or re-certification requires at least 80% agreement on three of the four FIMP annual test spots. In addition to inter-rater agreement, each site calculates an intraclass correlation (ICC) using criteria defined by Cicchetti [43]; this score is expected to be at or above 0.65 for the given year. Each coder’s ICC is calculated from the FIMP spots selected for annual fidelity testing excluding the lowest score. The lowest score refers to the FIMP coder’s lowest reliability with the consensus established by the group. This practice of dropping the lowest score, which is followed by all FIMP teams, creates room for coders to have one bad day while maintaining high performance expectations. When coders fail to achieve reliability on two consecutive occasions or when they fail the annual reliability test, retraining sessions are held. Such reliability issues occur occasionally in all teams. Unfortunately, we do not have data on the number of times this takes place, which is a limitation of this research.

For the ISII-1 Team, the Fidelity Director guides the discussion until a consensus score is reached. The intersection of the participants’ diverse roles makes for dynamic discussion that energizes the model’s guardians of fidelity to maintain reliability and prevent drift across sites. Detailed notes from each meeting provide a record of the process and are available, post-scoring, to FIMP team leaders during the annual reliability test. The ISII-1 Team reviews approximately 10 FIMP spots per year and selects spots with high consensus that can be used for the coming year’s annual FIMP test, which is taken by the implementation site teams. Site teams rate four spots from this library of FIMP spots. If they do not achieve reliability, they are assigned another spot from the pool. See the FIMP manual [11] for further information.

## Results

In this article, we provide FIMP team inter-rater reliability data in terms of percent agreement and ICC scores and meeting attendance. ICC estimates and their 95% confidence intervals were calculated using SPSS v 28.0, based on a mean rating (*k* = 2), absolute agreement, 2-way mixed-effects model. Data for five years (2016–2020) are for the purveyor’s two FIMP teams and the seven independent implementation site teams. Statistical analyses were carried out in the SPSS v 28.0.

### ISII-1 Team fidelity

Table 1 summarizes inter-rater agreement scores and attendance for each FIMP coder over the five years under study. Every year, the ISII-1 Team met 11 times. The end of year meeting (December) is typically used to review scores for the year. The team scored one session segment per meeting for 10 meetings, with one exception in 2019 when the team scored only 9 segments. The team scored a total of 49 session segments during their fidelity meetings across the five years. Meeting attendance was excellent, between 40 (74.07%) to 53 (98.15%) with a mean attendance of 47.80 meetings (*SD* = 4.76). Table 1 shows each coder’s participation in meetings, number of segments coded, the number and percentage of ‘hits’ (i.e., times each coder achieved agreement with consensus in the 80–100% range), and attendance.

**Table 1:** ISII-1 Team: Number of coded segments, inter-rater percent agreement, and attendance

	Coder Agreement			Attendance	
Coder	*N* Segments Coded	*N* Hit	% Hit	*N*	%
Coder 1	46	43	93.48%	48	88.89%
Coder 2	48	47	97.92%	53	98.15%
Coder 3	46	44	95.65%	49	90.74%
Coder 4	41	36	87.80%	40	74.07%
Coder 5	45	40	88.89%	49	90.74%

Total possible segments = 49, total possible meetings = 54. The coder designation (e.g., Coder 1) is a pseudonym. Hit refers to agreement with the consensus score

The ISII-1 Team is responsible for selecting the FIMP spots that ISII-2 and the implementation sites use in their annual tests. Every year, the team viewed all possible spots and selected 6 to 10 spots to make available for annual fidelity testing. Table 2 shows the number of spots selected for testing each year as well as each fidelity coder’s ICC score. Data reveal that individual consensus team members met and exceeded the 0.65 cutoff with ICCs ranging from 0.700 to 0.936.

**Table 2:** ISII-1 Team: ICC scores and approved test spots

	Rating Year				
Coder	2016 (*n* = 7^a^)	2017 (*n* = 8)	2018 (*n* = 9)	2019 (*n* = 5)	2020 (*n* = 6)
Coder 1	.886	.831	.864	.831	.789
	(*n* = 5)	(*n* = 8)	(*n* = 9)	(*n* = 4)	(*n* = 6)
Coder 2	.866	.869	.854	.927	.768
	(*n* = 7)	(*n* = 8)	(*n* = 8)	(*n* = 5)	(*n* = 6)
Coder 3	.712	.936	.894	.882	.801
	(*n* = 6)	(*n* = 7)	(*n* = 8)	(*n* = 4)	(*n* = 6)
Coder 4	.732	.879	.831	.700	.727
	(*n* = 7)	(*n* = 7)	(*n* = 7)	(*n* = 4)	(*n* = 6)
Coder 5	.852	.848	.918	.749	.812
	(*n* = 7)	(*n* = 7)	(*n* = 7)	(*n* = 5)	(*n* = 6)
Total Approved Spots	8	9	10	6	7

The coder designation represents the same coders in Table 1. ICC scores represent a coder’s ratings minus their lowest score. The number of approved spots varies due to coders’ attendance. For example, Coder 1 attended nine meetings of 11 meetings in 2016, but was only present at 6 of the 8 meetings where spots were rated and approved for testing

^a^This value represents the number of approved spots minus one. The lowest score is dropped for each coder. For example, in 2016 there were 8 possible test spots; for a coder with perfect attendance, ICCs were calculated on their 7 best scores

Numbers of approved test spots dropped in 2019 and 2020. That was because in May of 2018, the new General Data Protection Regulation (GDPR) went into effect. The GDPR has strict standards for data protection for all countries in the European Union (gdpr-info.eu). This affected access to video segments from our European sites. The COVID-19 pandemic also affected availability in 2020.

### Implementation Sites and ISII-2 fidelity monitoring

Table 3 includes the data for the ISII-2 team (nicknamed Alpha) and the seven implementation sites for a total of eight fidelity teams. Across all teams, there were 51 FIMP coders. Attendance data were available for nearly all fidelity teams. Sites held 8 to 11 meetings per year. While attendance varied tremendously (from 0 to 100%), the mean attendance across sites for each year ranged from 79.27% to 90.28%, signaling strong attendance overall. Across the eight coding teams, there were 37 meetings in 2016, 49 in 2017, 59 in 2018, 58 in 2019, and 64 in 2020.

**Table 3:** ISII-2 and independent implementation site teams: coder numbers and ICC ranges for 2016 to 2020

Site	FIMP Coders 2016	ICC Ranges	FIMP Coders 2017	ICC Ranges	FIMP Coders 2018	ICC Ranges	FIMP Coders 2019	ICC Ranges	FIMP Coders 2020	ICC Ranges
Alpha	6	.62—.88	5	.80—.92	5	.67—.80	5	.79—.91	6	.64—.87
Beta	8	.74—86	8	.60—.87	6	.78—.88	6	.65—.84	3	.71—.83
Gamma	5	.62—.91	4	.70—.93	5	.77—.96	5	.66—.88	5	.67—.94
Delta	8	.85—.95	8	.83—.90	6	.82—.91	7	.80—.94	5	.82—.93
Epsilon	7	.62—.95	13	.64—.88	11	.70—.92	15	.61—.93	10	.68—.89
Zeta	-	-	13	.59—.94	13	.72—.94	-	-	12	.77—.95
Eta	-	-	-	-	3	.62—.76	3	.74—.90	4	.74—.94
Theta	-	-	-	-	-	-	-	-	4	.69—.85
Total	34	.62—.95	51	.59—.94	49	.62—.96	41	.61—.94	49	.64—.95

The ISII-2 Team is mentioned first and labeled as Alpha. Eta became an independent FIMP team in 2018 and Theta in 2020. Data is missing from Zeta in 2016 and 2019

Table 3 provides coders and ICCs across sites for each of the five years. A review of data shows strong reliability across and within teams. The ICC standards of 0.65 or above were stringent. We followed Cicchetti’s [43] guidelines for inter-rater agreement which specified 0.60—0.74 as “good” and 0.75 to 1.00 as “excellent”. The scores across five years and eight teams were at or above the 0.65 cutoff for 96.35% of all coders. In 2016, 91.18% or 31 of 34 coders met the cutoff. The three coders that did not meet the cutoff were all at 0.62. In 2017, 94.12% (48 of 51) of coders met the cutoff. The three coders that did not meet criteria scores 0.59, 0.60, and 0.64. In 2018, 97.95% (48 of 49) of coders scored above the cutoff. The remaining coder scored 0.62. In 2019, 97.56% (40 of 41) of coders were reliable at 0.65 or higher. Only one coder fell below the range at 0.61. In 2020, 98.03% (50 of 51) were above the cutoff with the remaining coder scoring 0.64. According to Cicchetti’s criteria, all but one coder met the 0.60 cutoff for “good” reliability. A great majority of coders fell in the “excellent” range, specifically, 85.29% (*n* = 29) in 2016, 76.47% (*n* = 39) in 2017, 85.71% (*n* = 42) in 2018, 70.73% (*n* = 29) in 2019, and 78.43% (*n* = 40) in 2020.

## Discussion

This article presents inter-rater data for the Fidelity of Implementation Rating System (FIMP) [11], an assessment tool that monitors fidelity following widescale implementation of the GenerationPMTO model [9]. We describe the painstaking process through which the construct validity of the measurement tool is maintained (e.g., monthly fidelity meetings of the ISII-1 team that also often include implementation site FIMP leads) and provide evidence that meeting attendance and reliability for the selected FIMP spots is strong across seven international implementation sites. The findings indicate that this observation-based system can be employed to maintain reliable ratings by implementation sites post implementation. The ISII-1 Team achieved stable levels of good to excellent inter-rater reliability and ICC scores and maintained good attendance at fidelity meetings evidencing strong commitment to maintaining construct validity. Data for the implementation sites and the ISII-2 team mirrored findings for the ISII-1 Team: reliability scores were good to excellent with only a few coders not meeting the cut-off over the years, and meeting attendance was good. The answer to both our research questions is therefore positive.

For the ISII-1 team, regular meetings include discussion of the model and its application across sites while addressing issues with fidelity and implementation. The ISII Fidelity Director is the lynchpin between the ISII-1 team and site leaders to ensure that the consensus reached reflects international standards while taking local contexts into account. Meetings within the sites foster engagement and connection to the model and to each other as a team. The inclusion of site leads in the ISII-1 fidelity meetings provides an opportunity for direct discussion and fidelity consensus building between the site and ISII; these discussions, in turn, are fed back to the sites to ensure their access to ongoing discussions, decisions, and updates to the FIMP manual. The process sharpens the leaders’ lens when assessing fidelity based on observations of intervention, training, coaching, and certification, while promoting sustained fidelity to the model.

The use of coded observations or audiotapes of sessions to score fidelity is now considered gold standard [44–46], with growing evidence for the predictive validity and reliability of these measures across time and coders [22–25, 37, 41]. To our best knowledge, there are few well-documented examples of systems that monitor and sustain reliable fidelity ratings in real-world settings years following implementation. This is particularly true for measures based on direct observation. One likely reason for this dearth of research is the intensive work required for this type of assessment.

Direct observation has been criticized as time consuming, costly, and difficult to do in community practice [3, 7]. The pressure to adopt methods that require less time and effort begs the question: how much of an intervention session needs to be observed in order to accurately assess fidelity? In the FIMP system, fidelity is assessed by coding 10 to15 minute spots of sessions on the delivery of two core components, encouragement, and limit setting. Fidelity on delivery of these two components has shown to predict improved parenting outcomes [23, 25] as well as improved pre/post child outcomes [24].

Extending coder reliability to other components could address complex research questions and identify other intervention components as active ingredients that produce positive outcomes. An example of this was found in a study by Holtrop et al. [47]. They observed and fidelity rated 89% of all parent group sessions in a GenerationPMTO study with recently separated mothers [48] using the Component Level Implementation Fidelity Rating System/CLIFRS, a fidelity system developed by Holtrop and colleagues. They found that extent of delivery of four intervention components during group sessions (emotion regulation, effective communication, problem solving and monitoring) was associated with pre/post reduction in observed coercive parenting. Emotion regulation and effective communication are not identified as core components currently. Should they be elevated to core component status?

Harder to capture empirically but crucially important is how FIMP ratings are affected by contextual, linguistic, and cultural factors, and the ways they evolve in response to societal and generational change. To address this, the ISII-1 Team members, who have their own cultural backgrounds, invite FIMP leaders from different countries and cultural backgrounds to participate in meetings when a spot from their sites is scored. The variety and richness of material from across cultures and countries help the consensus team focus on the core of the model across different contexts. Because these discussions are based on observation, it becomes clear how both competence and adherence translate to real world settings and where drift may occur in fidelity across sites. As with any group of leaders, strong opinions could obliterate hope for agreement, but that threat is rendered moot due to skillful leadership by the Fidelity Director, who joins, acknowledges perspectives, keeps the team focused on scoring-by-the-manual, and deftly ensures that all voices are heard in order to help the team reach consensus (masked, personal communication). These discussions deepen the understanding of the model and provide ideas for further development of the model and the rating system. This process helps visiting site fidelity leaders support accurate fidelity assessment within their sites and strengthens relationships and collaboration.

Regular, frequent meetings seem necessary to maintain this focus and level of inter-rater reliability. This process may be similar to what Ericsson [49] and Miller and colleagues [50, 51] describe in their studies of what contributes to excellence: determining your baseline, constant feedback and deliberate practice lead to constant development. Lack of feedback and reflection lead to diminished performance [51].

A few societal factors have influenced the use of the fidelity system. In 2018, FIMP procedures were changed in response to the GDPR in Europe. This law prevents non-European sites from viewing European material although Europeans can view material from sites outside of the EU. To address this restriction, EU sites now view data from non-EU sites for their annual FIMP reliability tests. When they participate in ISII-1 meetings, they view a FIMP spot from a non-EU country. These restrictions have led to decreased variety in spots in the consensus team meetings as well as limiting the annual FIMP test to non-EU spots only. This in turn limits the collaboration across sites in reaching consensus on culturally diverse spots and narrows examples. This has led to illuminating experiments in which several European spots have been coded in the consensus team with US members reading a transcript and EU members having access to transcript and video. COVID has also contributed to fewer available spots, as well as a push to reach consensus on telehealth GenerationPMTO intervention formats. The ISII-1Team continues to discuss the core components and practices of PMTO, albeit within new contexts. ISII is planning for the future by seeking legal and governmental expertise in the US and EU on privacy protocol and collaborating with current and prospective implementation sites to find a solution. Additionally, FIMP spots from non-EU countries, including Chile and Canada, are available for rating.

### Limitations

As discussed above, fidelity systems that cross national boundaries using identifiable data (in this case, observations of intervention sessions) are subject to privacy regulations. Collaborating on a global scale in this manner requires access to each other’s data through safe online systems and legislation that makes this possible; that has been a challenge as noted above, especially in the last years. For the preparation of this manuscript, leaders from each site granted their support for the use and reporting of these deidentified data. Requiring that practitioners video record sessions implies the use of equipment and technology; furthermore, some families refuse. Uploading sessions to the portal can also be time consuming. A substantial investment in time and effort is needed for regular meetings and ratings of sessions, which can be seen as another limitation. In our experience it is worth it, and as far as we know there is not a well-established, more efficient alternative.

### Implications for research and practice

Future research could examine reliability within each site´s FIMP Team to see how that relates to reliability with the ISII-1 Team. Also, it will be important to broaden the scope of rating and reliability to other components to better understand the contribution of other potential effective intervention components. If data emerge showing that additional components contribute to positive outcomes, developers may need to include them as core components. Further research should identify the minimal time needed to optimally monitor fidelity in terms of session segments and number of ratings required for good reliability. Additional research could explore potential impacts on long-term sustainability, e.g., benefits of professional growth and development for sites’ FIMP team members, reciprocity across a site’s agencies (i.e., one agency provides fidelity rating in exchange for another agency providing coaching), and how sites balance belief in an EBPP’s ability to promote positive family change with the harsh realities of funding a non-billable expense.

## Conclusion

This study provides an example of a system that can safely transfer EBPPs to community settings while continually monitoring and maintaining fidelity over time. The results show that the program developer teams and implementation sites can maintain good to excellent levels of inter-rater reliability, ICC scores, and good attendance over time by following this structure. This study maps an efficient way forward in using observation-based data to monitor and strengthen fidelity in real world practice to ensure that families get the best possible treatment in community settings. The FIMP system has strengthened mutual understanding of the model through a shared language that is able to cross borders and transcend cultures. This common language based on observation has prevented things from being ‘lost in translation’ and helps prevent program drift. It has also greatly facilitated international collaboration and fostered long lasting relationships between the developers and the sites. This could be useful knowledge for other evidence-based programs in the field.

## Data Availability

Not applicable.

## Abbreviations

CLIFRS: Component Level Implementation Fidelity Rating System
EBPP: Evidence-based parenting program
FCU: Family Check Up
FIMP: Fidelity of Implementation rating system
GDPR: General Data Protection Regulation
ICC: Intraclass correlation
ISII: Implementation Sciences International, Inc
PMTO: Parent Management Training – Oregon Model
OSLC: Oregon Social Learning Center
